# A systematic review evaluating loneliness assessment instruments in older adults

**DOI:** 10.3389/fpsyg.2023.1101462

**Published:** 2023-04-25

**Authors:** César Bugallo-Carrera, Carlos Dosil-Díaz, Luis Anido-Rifón, Moisés Pacheco-Lorenzo, Manuel J. Fernández-Iglesias, Manuel Gandoy-Crego

**Affiliations:** ^1^Department of Developmental Psychology, University of Santiago de Compostela, Santiago de Compostela, Spain; ^2^AtlanTTic Research Center, University of Vigo, Vigo, Spain; ^3^Department of Psychiatry, Radiology, Public Health, Nursing and Medicine, University of Santiago de Compostela, Santiago de Compostela, Spain

**Keywords:** solitude, loneliness, older adults, geriatric, loneliness assessment tools

## Abstract

**Introduction and objectives:**

The experiences and changes that come along with old age may lead to a feeling of loneliness, usually followed by negative physical and mental manifestations. In this systematic review, we evaluated the existing tools to assess loneliness in older adults.

**Methods:**

We performed a literature search in the Web of Science, Medline, and PsycINFO, following the Preferred Reporting Items for Systematic Reviews and Meta-Analyses (PRISMA) guidelines. After, we examined the psychometric properties of the instruments with a focus on reliability, validity, and main conclusions.

**Results:**

We included 27 articles published between 1996 and 2021.

**Conclusion:**

To date, there are few instruments to assess loneliness in older adults. In general, they present adequate psychometric properties, although it is true that some scales show somewhat low levels of reliability and validity.

## Introduction

There are common situations and changes experienced by people during the process of aging such as chronic conditions, declining physical function, widowhood, or retirement (Cohen-Mansfield et al., 2016; Ong et al., 2016). These may promote a feeling of loneliness—understood as a subjective negative experience—determined by a cognitive assessment in which there are discrepancies between the social relationships older individuals want and those they truly possess (De Jong Gierveld et al., 2015). Inevitably, the person experiences a painful feeling when they perceive a lack of social relationships (unsatisfactory or inadmissible), either because the number of contacts is lower than they would want or because the relationships are not as intimate as expected (De Jong-Gierveld, 1987).

However, loneliness does not always lead to negative feelings, as in the case of objective loneliness, i.e., a lack of company that does not necessarily imply an unpleasant experience for the older adult, which may even be sought and fulfilling. In contrast, in subjective loneliness, the individual feels alone, and this causes unsought feelings of pain (Rodríguez Martín, 2009). This dichotomous view of loneliness goes back to the 1970s, when Moustakas (1972) distinguished between “loneliness,” i.e., loneliness as an unpleasant and unwanted experience, and “solitude,” i.e., chosen loneliness.

The prevalence of loneliness varies widely among older adults, from 9% in the UK (Victor and Bowling, 2012) to 60.2% in Taiwan (Wang et al., 2001). This variability may be explained by factors such as interindividual differences, used tools or research methods, or dissimilarities in cultural and social backgrounds (Huang et al., 2021).

Loneliness and the negative feeling that usually accompanies it have unfavorable physical and mental consequences on the individual (Sánchez and Bote, 2007). Different studies have shown that loneliness increases the likelihood of anxiety, depression, and sleep disorders (Sánchez and Bote, 2007; Portellano-Ortiz et al., 2016; Acosta et al., 2017); it also increases the risk of having chronic diseases (cardiovascular disease, hypertension, stroke, pulmonary disease, obesity, and/or diabetes; Petitte et al., 2015), increases the risk of developing neurodegenerative conditions (e.g., Alzheimer’s disease; Boss et al., 2015; Donovan et al., 2016), and significantly increases the likelihood of suicide (Sancho Castiello, 2012).

Moreover, loneliness in the 21st century has its own characteristics. On the one hand, some sociodemographic elements affect loneliness, e.g., changes in family structure or current family models associated with lower birth rates, the crisis in the care system, lack of family protection, and increased widowhood, longevity, and life expectancy. On the other hand, loneliness is increasing with the shift from a mainly rural society, where coexistence and interpersonal relationships are easier, to an urban society, where personal encounters become more complex. Furthermore, digital communication prevents the forging of personal connections. Interpersonal (face-to-face) communication helps to develop relations to get rid of loneliness. Last but not least, there has been an increase of frequently imposed institutionalization of people of an older age, and these individuals become socially isolated when taken away from the environment they have built for decades (Lobo, 2020).

Properly validated tools to detect individuals at risk of suffering loneliness are key, as they would allow intervening as soon as possible to help prevent the associated negative consequences of loneliness and to assess the effectiveness of potential therapies.

The aim of this systematic review was to analyze the currently available validated instruments that assess loneliness in aged individuals and to determine if there is a sufficient number of them and their suitability.

## Methods

We conducted a systematic review following the Preferred Reporting Items for Systematic Reviews and Meta-Analyses (PRISMA) guidelines (Page et al., 2021). Next, we examined the psychometric properties of the tools—focusing on reliability and validity—and collected the main results and/or conclusions.

We carried out the search through the Web of Science, Medline, and PsycINFO, including articles published until February 2022 and using the following combinations of terms and Boolean operators: AND OR: ((ALL = (elderly OR older) AND ALL = (“measuring loneliness” OR “measure of loneliness” OR “assessing loneliness” OR “loneliness assessment” OR “loneliness scale”)) AND ALL = (validity OR validation). The search was limited to studies with participants older than 65 years of age and written in English, Spanish, Portuguese, or French.

We obtained 279 potential articles in our search (Web of Science: 151; Medline: 22; PsycInfo: 106).

All the registries were combined in a single file to identify duplicates after a manual check, after which 231 potential articles remained. Next, we analyzed the titles and abstracts; at this step, we excluded 176 articles. A total of 49 did not validate a tool, and 18 did not include older people, or the age group was not adequately stratified. In 108 of them, loneliness was not the main objective of the study, and 1 was not an article.

After the above-described pre-selection steps, we further reviewed 55 articles that met the eligibility criteria. After reading these articles in full, 10 were removed either because a tool was not validated or not validated in a context of normality, 14 did not include older participants or the age group was not adequately stratified, 3 because loneliness was not the main topic of the study, and 1 was not an article.

Inclusion criteria:

- Empirical research; not reviews, single-case studies, books, nor manuals.
- Studies that validated a tool within a context of normality.
- Studies that addressed loneliness as the main objective.
- Studies focused on older adults or in which this age group was adequately stratified.
- Written in English, Spanish, Portuguese, or French.

Exclusion criteria:

- Non-empirical research.
- Studies that did not validate a tool within a context of normality.
- Studies that did not address loneliness as the main objective.
- Studies not focused on older adults or in which this age group was not adequately stratified.
- Studies written in languages other than English, Spanish, Portuguese, or French (Supplementary Figure 1).

## Results

Twenty-seven articles published between 1996 and 2021 were included in this systematic review, including 23 written in English and 4 in Spanish, although 28 validations were carried out as two instruments were validated in one of the works (Penning et al., 2014).

Fourteen validations of some of the versions of the De Jong Gierveld Loneliness Scale (DJGLS; De Jong-Gierveld and Kamphuls, 1985) were carried out; specifically, eight validations of the 6-item DJGLS (Leung et al., 2008; De Jong Gierveld and Van Tilburg, 2010; Ayala et al., 2012; Iecovich, 2013; Cheung et al., 2020; Hosseinabadi et al., 2020; Jaafar et al., 2020; Rodríguez-Blázquez et al., 2021) and six validations of the 11-item DJGLS (Buz and Pérez-Arechaederra, 2014; Penning et al., 2014; Tomás et al., 2017; Uysal-Bozkir et al., 2017; Caycho-Rodríguez et al., 2021; Hosseinabadi et al., 2021). In the study by De Jong Gierveld and Van Tilburg (2010), the validated emotional DJGLS-3 and social DJGLS-3 scales are equivalent to the DJGLS-6 scale.

Eleven validations of some of the versions of the University of California Los Angeles (UCLA) Loneliness Scale were performed (Russell et al., 1978): one corresponding to the 20-item UCLA Loneliness Scale (Park et al., 2019), one to the 10-item UCLA Loneliness Scale (Velarde-Mayol et al., 2016), three to the 20-item UCLA-R (Penning et al., 2014; Ausín et al., 2019; Lee et al., 2021), one to the 11-item UCLA-R (Lee and Cagle, 2017), three to the 20-item UCLA-3 (Russell, 1996; Durak and Senol-Durak, 2010; Sancho et al., 2020), one to the 6-item ULS-6 (Neto, 2014), and one to the 16-item ULS-16 (Faustino et al., 2019).

Moreover, there was one validation of the 22-item Loneliness Literacy Scale (LLS; Honigh-de Vlaming et al., 2014), one validation of the 34-item *ESTE* scale (González-Tovar and Garza-Sánchez, 2021), and one of the 3-item Loneliness (TIL) scale (Pedroso-Chaparro et al., 2021; Supplementary Table 1).

We analyzed the internal reliability of the instruments by estimating Cronbach’s alpha, except in the works by Tomás et al. (2017) (CRI constructor), Rodríguez-Blázquez et al. (2021) (Kuder–Richardson formula 20: KR-20), and González-Tovar and Garza-Sánchez (2021) (McDonald’s omega coefficient). Thus, 17 studies show good internal reliability (≥0.8; Russell, 1996; De Jong Gierveld and Van Tilburg, 2010; Durak and Senol-Durak, 2010; Iecovich, 2013; Honigh-de Vlaming et al., 2014; Neto, 2014; Penning et al., 2014; Velarde-Mayol et al., 2016; Lee and Cagle, 2017; Tomás et al., 2017; Uysal-Bozkir et al., 2017; Ausín et al., 2019; Faustino et al., 2019; Park et al., 2019; Caycho-Rodríguez et al., 2021; González-Tovar and Garza-Sánchez, 2021; Lee et al., 2021).

The intraclass correlation coefficient (ICC) was reported in the studies by Leung et al. (2008), Neto (2014), and Hosseinabadi et al. (2020) and test–retest reliability measures in the work by Jaafar et al. (2020).

The construct validity was reported in all the studies except in one (Jaafar et al., 2020). The dimensionality of the instruments using factor analysis was analyzed in 21 studies (Russell, 1996; De Jong Gierveld and Van Tilburg, 2010; Durak and Senol-Durak, 2010; Ayala et al., 2012; Iecovich, 2013; Neto, 2014; Penning et al., 2014; Velarde-Mayol et al., 2016; Lee and Cagle, 2017; Tomás et al., 2017; Uysal-Bozkir et al., 2017; Ausín et al., 2019; Faustino et al., 2019; Cheung et al., 2020; Hosseinabadi et al., 2020, 2021; Caycho-Rodríguez et al., 2021; González-Tovar and Garza-Sánchez, 2021; Lee et al., 2021; Pedroso-Chaparro et al., 2021; Rodríguez-Blázquez et al., 2021); the Delphi method in one study (Leung et al., 2008); principal component analysis without rotation in 1 study (Buz and Pérez-Arechaederra, 2014); principal component analysis with oblique rotation in 1 study (Honigh-de Vlaming et al., 2014); the Rasch model in 1 study (Park et al., 2019); and exploratory structural equation modeling in 1 study (Sancho et al., 2020). Overall, the instruments are multidimensional, and thus so is loneliness, except for seven studies that conclude that the instruments are unidimensional (Russell, 1996; Buz and Pérez-Arechaederra, 2014; Neto, 2014; Velarde-Mayol et al., 2016; Tomás et al., 2017; Pedroso-Chaparro et al., 2021; Rodríguez-Blázquez et al., 2021).

The convergent validity was examined in 12 studies (Russell, 1996; Durak and Senol-Durak, 2010; Iecovich, 2013; Buz and Pérez-Arechaederra, 2014; Neto, 2014; Tomás et al., 2017; Faustino et al., 2019; Jaafar et al., 2020; Sancho et al., 2020; Caycho-Rodríguez et al., 2021; Pedroso-Chaparro et al., 2021; Rodríguez-Blázquez et al., 2021), content validity in 3 studies (Leung et al., 2008; Hosseinabadi et al., 2020, 2021), and discriminant validity was reported in 1 study (Velarde-Mayol et al., 2016).

## Discussion

In our search, we initially identified 279 registries, 27 of which (published between 1996 and 2021) meet the established inclusion criteria. To date, many instruments to assess loneliness in older adults have been validated. The fact that we detected only 27 studies is indicative of the traditional lack of interest in studying a difficult-to-detect and little-known phenomenon such as loneliness in adulthood. It is fair to mention that over the past years a larger number of this type of studies has been published.

A series of works have associated the negative feelings that accompany unwanted loneliness with the development or worsening of pathologies (Sánchez and Bote, 2007; Boss et al., 2015; Petitte et al., 2015; Donovan et al., 2016; Portellano-Ortiz et al., 2016; Acosta et al., 2017) or the increase in suicide rates in this age group (Sancho Castiello, 2012), which possibly explains the recent interest in the validation of these instruments.

From the 28 validations, 13 focus on different versions of the DJGLS (De Jong-Gierveld and Kamphuls, 1985), 11 on the different versions of the UCLA Loneliness Scale (Russell et al., 1978), 1 validated a version of the DJGLS and 1 of the versions of the UCLA Loneliness Scale, 1 centered the attention on the LLS (Honigh-de Vlaming et al., 2014), one on the *ESTE* (Rubio and Aleixandre, 1999), and 1 on the TIL (Hughes et al., 2004). Overall, the DJGLS and the UCLA DJGLS and la UCLA, both in their different versions, are the most widely used scales to assess loneliness in older adults.

Four studies show the transcultural validity of the DJGLS (De Jong Gierveld and Van Tilburg, 2010; Uysal-Bozkir et al., 2017; Cheung et al., 2020; Rodríguez-Blázquez et al., 2021) and one of the UCLA Loneliness Scale (Park et al., 2019).

Some outcomes of the different analyzed studies are contradictory, i.e., in some of the studies there are no sex-related differences regarding the effects of unsought loneliness (Leung et al., 2008; Neto, 2014; Pedroso-Chaparro et al., 2021; Rodríguez-Blázquez et al., 2021), while other studies report differences (De Jong Gierveld and Van Tilburg, 2010; Jaafar et al., 2020). Similar observations are seen for age: some studies find no association (Leung et al., 2008; Uysal-Bozkir et al., 2017; Pedroso-Chaparro et al., 2021; Rodríguez-Blázquez et al., 2021) and other do, specifying that the older the individual the greater the perceived feeling of loneliness (De Jong Gierveld and Van Tilburg, 2010; Neto, 2014). The latter findings are in line with other studies that report a positive relationship between age and loneliness (Victor and Yang, 2012; Nicolaisen and Thorsen, 2017).

Most studies examined in this systematic review agree there is a direct relationship between marital status and cohabitation with unsought loneliness; that is, the feeling of loneliness is higher in widows/widowers, divorcees, singles, and individuals living alone (Leung et al., 2008; De Jong Gierveld and Van Tilburg, 2010; Ayala et al., 2012; Buz and Pérez-Arechaederra, 2014; Neto, 2014; Velarde-Mayol et al., 2016; Uysal-Bozkir et al., 2017; Jaafar et al., 2020). This is in line with the works that support the existence of a strong association of widowhood and living alone with the feeling of loneliness (Dykstra et al., 2005; Sundström et al., 2009; Aartsen and Jylhä, 2011; De Jong Gierveld et al., 2012; Victor and Bowling, 2012; Lykes and Kemmelmeier, 2014).

In summary, this review work shows us the variety of validated tests currently available to assess loneliness in older people and the growing social and research interest on this subject in different cultures. Likewise, it also reflects that most of the available instruments have, globally, adequate psychometric properties, although it is true that some scales show somewhat low levels of reliability and validity; therefore, they could be improved.

Future research should focus on adapting scales involving different cultures and following a strict methodology regarding participant inclusion/exclusion, instrument administration protocol, transcultural validation considering the linguistic and symbolic adaptation of the meaning of the construct *loneliness* that is shared by the target population to be assessed, and the development of parallel versions of the instruments to help reduce the risk of contaminating repetitive evaluations (practice effect). Moreover, future researchers should be able to perform follow-ups of the population at risk of suffering unsought loneliness.

## Author contributions

MG-C, MF-I, MP-L, and LA-R designed the revision. CD-D and CB-C collected the data. CB-C analyzed the data. CB-C, CD-D, LA-R, MP-L, MF-I, and MG-C wrote the paper. All authors contributed to the article and approved the submitted version.

## Funding

This work has been partially funded by Ministerio de Ciencia e Innovación, project SAPIENS- Services and applications for a healthy aging (Grant PID2020-115137RB-I00 funded by MCIN/AEI/10.13039/501100011033).

## Conflict of interest

The authors declare that the research was conducted in the absence of any commercial or financial relationships that could be construed as a potential conflict of interest.

## Publisher’s note

All claims expressed in this article are solely those of the authors and do not necessarily represent those of their affiliated organizations, or those of the publisher, the editors and the reviewers. Any product that may be evaluated in this article, or claim that may be made by its manufacturer, is not guaranteed or endorsed by the publisher.

## Supplementary material

The Supplementary material for this article can be found online at: https://www.frontiersin.org/articles/10.3389/fpsyg.2023.1101462/full#supplementary-material
