# Supplementary material for: A systematic review evaluating loneliness assessment instruments in older adults
Source: Front Psychol. 2023 Apr 25;14:1101462. doi: 10.3389/fpsyg.2023.1101462 (PMC10166865; doi:10.3389/fpsyg.2023.1101462)
Supplement: Supplementary file 1 [file Table_1.docx]

| **Author, Year, Country** | **Instrument Participants** | | | | | | | | | | | | | | | | | | **Results, Conclusions** | | | | | | | | | | | | | |
| --- | --- | --- | --- | --- | --- | --- | --- | --- | --- | --- | --- | --- | --- | --- | --- | --- | --- | --- | --- | --- | --- | --- | --- | --- | --- | --- | --- | --- | --- | --- | --- | --- |
| Russell, 1996,  USA | UCLA-3 | Community sample  N  Sex (%, female)  Age | | 301  60  ˃ 65 years | | | | | | | | | | | | | | | Unidimensional (CFA)  Internal reliability  α=.89  Convergent validity  NYU Loneliness Scale: *r*=.65  Differential Loneliness Scale: *r*=.72  Social Provisions Scale: *r*=-.68  Social Desirability: *r*=-.21 | | | | | | | | | | | | | |
| Leung *et al.*, 2008,  China | DJGLS-6 | Community sample  N  Sex (%, female)  Age (M)    Education (years)  Exclusion criteria | | 103  49  74.5±6.5  4.8±4.9  Dementia (CDR) | | | | | | | | | | | | | | | Multidimensional (Delphi method)  Internal reliability  α=.76  ICC reliability  .98-1.00  Content validity  High  Relationship + without a partner and living alone  No relationship with age or sex  No relationship with education | | | | | | | | | | | | | |
| **Author, Year, Country** | **Instrument Participants** | | | | | | | | | | | | | | | | | | **Results, Conclusions** | | | | | | | | | | | | | |
| De Jong Gierveld, and Van Tilburg, 2010,  France, Germany, Netherlands, Russia, Bulgaria, Georgia, and Japan | Emotional DJGLS-3  Social DJGLS-3 | Community sample Multidimensional  *Sub-samples* Internal reliability (Cronbach’s alpha) | | | | | | | | | | | | | | | | | | | | | | | | | | | | | | |
|  |  | N  Sex (%, female)  Age (M)  With a partner (%) | | F  2541  55  68.7  58 | | G  2560  50  68.2  62 | | N  1565  56  68.0  58 | | | | R  2804  69  68.9  43 | B  2470  50  68.1  67 | | G  2266  60  69.2  58 | | J  1891  50  64.3  85 | | EL  SL | | F  .81  .85 | G  .85  .91 | | N  .88  .88 | | | R  .86  .88 | B  .91  .95 | | G  .87  .90 | | J  .86  .90 |
|  |  |  |  |  |  |  |  |  |  |  |  |  |  |  |  |  |  |  | Having a partner is important to relieve EL and SL to a lesser degree  Not having children leads to greater risk of SL  EL > in female  SL > in male  At older ages greater EL and SL  Reliable and valid for use in different countries | | | | | | | | | | | | | |
| Durak and Senol-Durak, 2010,  Turkey | UCLA-3 | Community sample  N  Sex (%, female)  Age (M) | | 166  55.4  63.70±5.31 | | | | | | | | | | | | | | | Multidimensional  Internal reliability  GL: α= .90  NL: α= .85  L: α= .84  Convergent validity  Geriatric depression: *r*=.59  Satisfaction with life: *r*=-.25  Self-esteem: *r*=-.46  Perceived health: *r*=-.34 | | | | | | | | | | | | | |
| **Author, Year, Country** | **Instrument Participants** | | | | | | | | | | | | | | | | | | **Results, Conclusions** | | | | | | | | | | | | | |
| Ayala *et al*., 2012,  Spain | DJGLS-6 | Community sample  N  Sex (%, female)  Age (M) | | 1106  56.3  72.07±7.83 | | | | | | | | | | | | | | | Multidimensional  Internal reliability  L: α= .77  EL: α= .58  SL: α= .87  Differences based on marital status, way of life, and disability | | | | | | | | | | | | | |
| Iecovich, 2013,  Israel | DJGLS-6 | Community sample  N  Sex (%, female)  Age (M) | | 1327  63.2  78.77±8.05 | | | | | | | | | | | | | | | Multidimensional  Internal reliability  S: α= .86  EL: α=.83  SL: α=.77  Convergent validity  WHOQOL-BREF: *r*=.65  SWLS: *r*=.66  VOL: *r*=.66 | | | | | | | | | | | | | |
| **Author, Year, Country** | **Instrument Participants** | | | | | | | | | | | | | | | | | | **Results, Conclusions** | | | | | | | | | | | | | |
| Penning *et al.*, 2013,  Canada | R-UCLA  DJGLS-11 | Community sample  N  Sex (%, female)  Age (range) | | 243  53.5  45-84 | | | | | | | | | | | | | | | R-UCLA  Multidimensional  Internal reliability  Factor 1: α= .87  Factor 2: α=.90  Factor 3: α=.86  Factor 4: α=.78 | | | | | | | | DJGLS-11  Multidimensional  Internal reliability  Factor 1: α=.87  Factor 2: α=.86 | | | | | |
|  |  |  |  |  |  |  |  |  |  |  |  |  |  |  |  |  |  |  | DJGLS-11 better than ç R-UCLA for studies with mean aged and older adults | | | | | | | | | | | | | |
| Buz and Pérez-Arechaederra, 2014,  Spain | DJGLS-11 | Community sample  N  Sex (%, female)  Age (M) | | 585  53.5  74.1±7.7 | | | | | | | | | | | | | | | Unidimensional  Internal reliability  α=.71  Convergent validity  Self-rated loneliness: *r*=.51  GDS-8: *r*=.55  NA: *r*=.37  SWLS: *r*=-.40  PA: *r*=-.40 | | | | | | | | | | | | | |
| **Author, Year, Country** | **Instrument Participants** | | | | | | | | | | | | | | | | | | **Results, Conclusions** | | | | | | | | | | | | | |
| Honigh-de Vlaming *et al.*, 2014,  Netherlands | LLS | Community sample  N  Sex (%, female)  Age (M) | | 303  55  75.5±6.7 | | | | | | | | | | | | | | | Multidimensional  Internal reliability  Motivation: α= .87  Self-efficacy: α= .83  Perceived social support: α= .74  Subjective norm: α= .81 | | | | | | | | | | | | | |
| Neto, 2014,  Portugal | ULS-6 | Community sample  N  Sex (%, female)  Age (M) | | 1154  60.5  71.26±6.66 | | | | | | | | | | | | | | | Unidimensional  Internal reliability  α= .82  Total item correlation  .45-.60  ICC reliability  .43  Convergent validity  R-UCLA: *r*=.92  Self-esteem scale: *r*=-.66  NA: *r*=.47  SWLS: *r*=-.43  PA: *r*=-.56 | | | | | | | | | | | | | |
| **Author, Year, Country** | **Instrument Participants** | | | | | | | | | | | | | | | | | | **Results, Conclusions** | | | | | | | | | | | | | |
| Velarde-Mayol *et al.*, 2016,  Spain | UCLA | Community and clinical samples  N  Sex (%, female)  Age (M)  Living (alone, %) | | 120  82.7  70.7±8.2  23.3 | | | | | | | | | | | | | | | Unidimensional  Internal reliability  α= .95  Discriminant validity (AUC)  95% CI of .55 - .69  63.2% of individuals who live alone feel | | | | | | | | | | | | | |
| Lee and Cagle, 2017,  USA | R-UCLA | Community sample  N  Sex (%, female)  Age | | 3706  58  ˃ 65 years | | | | | | | | | | | | | | | Multidimensional  Internal reliability  α= .87 | | | | | | | | | | | | | |
| Tomás *et al.*, 2017,  Spain | DJGLS-11 | Community sample  N  Sex (%, female)  Age (M) | | 335  67.5  63.97±5.56 | | | | | | | | | | | | | | | Unidimensional  Internal reliability  CRI= .89  Convergent validity  UCLA-3: *r*=68  DUKE-UNC: *r*= -.66 | | | | | | | | | | | | | |
| **Author, Year, Country** | **Instrument Participants** | | | | | | | | | | | | | | | | | | **Results, Conclusions** | | | | | | | | | | | | | |
| Uysal-Bozkir *et al.*, 2017,  Netherlands | DJGLS-11 | Community sample  *Sub-samples* | | | | | | | | | | | | | | | | | Multidimensional  Internal reliability (Cronbach’s alpha) | | | | | | | | | | | | | |
|  |  | N  Sex (%, female)  Age (M) | | T.I.  215  55.8  63.8±6.7 | | | M.I.  138  31.2    63.7±6.5 | | | | S.I.  172  64.5    66.1±8 | | IS.I  65  61.5  65.4±7.8 | | | D  488  51.8  67.6±8.1 | | | L  EL  SL | | T.I.  .90  .85  .82 | | M.I.  .85  .73  .81 | | | S.I.  .92  .92  .82 | | IS.I  .87  .86  .78 | | | D  .87  .83  .81 | |
|  |  |  |  |  |  |  |  |  |  |  |  |  |  |  |  |  |  |  | Having a partner: protective factor against loneliness  Loneliness unrelated to age | | | | | | | | | | | | | |
| Ausín *et al.*, 2018,  Spain | R-UCLA | Community sample  N  Sex (%, female)  Age (M) | | 409  47  73.32±6.03 | | | | | | | | | | | | | | | Multidimensional  Internal reliability  α= .85 | | | | | | | | | | | | | |
| Faustino and others, 2018,  Portugal | ULS-16 | Community sample  N  Sex (%, female)  Age (M) | | 154  59.1  78.80±8.58 | | | | | | | | | | | | | | | Multidimensional  Internal reliability  Total: α= .93  Social isolation: α= .92  Affinities: α= .82  Convergent validity  MSPSS (family) - ULS (Social isolation): *r*= -.47  MSPSS (friends) - ULS (Social isolation): *r*= -.35 | | | | | | | | | | | | | |
| **Author, Year, Country** | **Instrument Participants** | | | | | | | | | | | | | | | | | | **Results, Conclusions** | | | | | | | | | | | | | |
|  |  |  | |  | | | | | | | | | | | | | | | MSPSS (family) - ULS (affinities): *r*= -.31  MSPSS (friends) - ULS (affinities): *r*= -.43  IADL - ULS (Social isolation): *r*= -.08  IADL - ULS (Affinities): *r*= .02 | | | | | | | | | | | | | |
| Park *et al*., 2019,  China and Korea | UCLA | Community sample  N (total) | | | 493  *Sub-samples* | | | | | | | | | | | | | | Multidimensional  Internal reliability  Isolation: α= .88  Relational connectedness: α= .87  Collective connectedness: α= .78  China´s scorings are higher for isolation and relational connectedness  Korea´s scorings are higher for collective connectedness | | | | | | | | | | | | | |
|  |  | N  Sex (%, female)  Age (M) | | | China  287  48.1  72.3±7.6 | | | | | Korean  206  69.9  78.1±7.8 | | | | | | | | |  |  |  |  |  |  |  |  |  |  |  |  |  |  |
| Sancho *et al*., 2019,  Spain | UCLA-3 | Community sample  N  Sex (%, female)  Age (M) | | | 335  67.5  63.97±5.56 | | | | | | | | | | | | | | Multidimensional  Internal reliability  Isolation: α= .88  Loneliness feature: α= .71  Social connectedness: α= .78  Convergent validity  DJGLS - Isolation: *r*= .64  DJGLS - Loneliness feature: *r*= .49  DJGLS - Social connectedness: *r*= .61  DUKE-UNC- Isolation: *r*= -.63  DUKE-UNC- Loneliness feature: *r*= -.42 | | | | | | | | | | | | | |
| **Author, Year, Country** | **Instrument Participants** | | | | | | | | | | | | | | | | | | **Results, Conclusions** | | | | | | | | | | | | | |
|  |  | | | | | | | | | | | | | | | | | | DUKE-UNC- Social connectedness: *r*= -.61 | | | | | | | | | | | | | |
| Cheung, Hobbelen *et al*., 2020,  China, Netherlands | DJGLS-6 | Community samples  N  Sex (%, female)  Age (M) | | | *Sub-samples* | | | | | | | | | | | | | | | Multidimensional    Internal reliability | | | | | | | | | | | | |
|  |  |  |  |  | China  193  53  70.3±6.5 | | | Chinese immigrants  135  59  70.7±8.8 | | | | | | | | | | | | L: α=  EL: α=  SL: α= | | China  .68  .61  .83 | | | Chinese immigrants  .71  .59  .81 | | | | | | | |
|  |  |  |  |  |  |  |  |  |  |  |  |  |  |  |  |  |  |  |  | China: lower load for item 3 (feeling of rejection)  Psychometric properties similar for both countries  Transcultural equivalency | | | | | | | | | | | | |
| Hosseinabadi *et al.*, 2020,  Iran | DJGLS-6 | Community sample  N  Sex (%, female)  Age (M) | | | 224  46.9  67.5±7.6 | | |  | | | | | | | | | | | | Multidimensional  Internal reliability  α=.69  ICC reliability  .74  Content validity  CVI= 0.87 | | | | | | | | | | | | |
| **Author, Year, Country** | **Instrument Participants** | | | | | | | | | | | | | | | | | | **Results, Conclusions** | | | | | | | | | | | | | |
| Jaafar *et al.*, 2020,  Malaysia | DJGLS-6 | Community sample  N  Sex (%, female)  Age  Marital status (married, %) | 200  56  > 50  87.5 | | | | | | | | | | | | | | | Internal reliability  α= .71  Test-retest reliability: *r*= .93  Convergent validity  ULS-8: *r*=.56  Differences in sex, ethnicity, area of residence, and marital status | | | | | | | | | | | | | | |
| Rodríguez-Blázquez *et al*., 2020,  Chile | DJGLS-6 | Community sample  N  Sex (%, female)  Age (M)  Education (%)  < primary school  Primary school  ≥ high school | *Sub-samples* | | | | | | | | | | | | | | | Unidimensional  Internal reliability (KR-20) | | | | | | | | | | | | | | |
|  |  |  | Non-indigenous  231  46  73.13±7.68  24.2  51.9  23.8 | | | | | | *Aymara*  201  53  70.85±7.81  63.2  24.4  12.4 | | | | | *Mapuche*  368  49  72.07±7.83  67.9  20.7  11.4 | | | |  |  |  |  |  |  |  |  |  |  |  |  |  |  |  |
|  |  |  |  |  |  |  |  |  |  |  |  |  |  |  |  |  |  | L:  SL:  EL: | | | | Non-indigenous  .71  .73  .62 | | | | | *Aymara*  .63  .86  .44 | | *Mapuche*  .65  .75  .44 | | | |
|  |  |  |  |  |  |  |  |  |  |  |  |  |  |  |  |  |  | Convergent validity | | | | | | | | | | | | | | |
|  |  |  |  |  |  |  |  |  |  |  |  |  |  |  |  |  |  | WHOQOL: *r*=  GDS-15: *r*=  BRCS: *r*=  PWI: *r*= | | | | Non-indigenous  -.47  .52  -.51  -.51 | | | | | *Aymara*  -.26  .34  -.29  -.29 | | *Mapuche*  -.47  .40  -.36  -.55 | | | |
|  |  | | | | | | | | | | | | | | | | | No differences in f(x) for age, sex, and ethnicity  Differences in f(x) for education only in non-indigenous | | | | | | | | | | | | | | |
| **Author, Year, Country** | **Instrument Participants** | | | | | | | | | | | | | | | | | | **Results, Conclusions** | | | | | | | | | | | | | |
| Caycho-Rodríguez *et al*., 2021,  Peru | DJGLS-11 | Community sample  N  Sex (%, female)  Age (M) | 235  84.7  72.69±6.68 | | | | | | | | | | | | | | | Multidimensional  Internal reliability  Total: α = .80  GLF: ω = .91  MF: ω = .79  Convergent validity  UCLA-3: *r*=.88  SWLS: *r*=-.71  BRCS: *r*=.76  PHQ-2: *r*=-.72 | | | | | | | | | | | | | | |
| Hosseinabadi *et al.*, 2021,  Iran | DJGLS-11 | Community sample  N  Sex (%, female)  Age (M) | 204  46.9  67.4±7.4 | | | | | | | | | | | | | | | Multidimensional  Internal reliability  α=.77  Content validity  CVI= .88 | | | | | | | | | | | | | | |
| **Author, Year, Country** | **Instrument Participants** | | | | | | | | | | | | | | | | | | **Results, Conclusions** | | | | | | | | | | | | | |
| González-Tovar, and Garza-Sánchez, 2021,  Mexico | ESTE | Community sample  N  Sex (%, female)  Age (M) | 396  55.8  69.81±6.96 | | | | | | | | | | | | | | | Multidimensional  Internal reliability  FL: ω= .66  ML: ω= .98  SL: ω= .88  EC: ω= .89  Total: ω= .92 | | | | | | | | | | | | | | |
| Lee *et al*., 2021,  Korea | R-UCLA | Community sample  N  Sex (%, female)  Age | 1041  77.6  77.69±5.52 | | | | | | | | | | | | | | | Multidimensional  Internal reliability  IL: α = .88  RL: α = .84 | | | | | | | | | | | | | | |
| Pedroso-Chaparro and others, 2021,  Spain | TIL | Community sample  N  Sex (%, female)  Age (M) | 314  70.70  71.98±6.16 | | | | | | | | | | | | | | | Unidimensional  Internal reliability  α =.78  Convergent validity  CES-D: *r*=.60  GAI: *r*=.51  No relation between loneliness and sex or age | | | | | | | | | | | | | | |

UCLA-3: University of California Los Angeles Scale (version 3); CFA: Confirmatory Factor Analysis; DJGLS-6: De Jong Gierveld 6-item Loneliness Scale; CDR: Clinical Dementia Rating; ICC: Intraclass Correlation Coefficient; DJGLS-3: De Jong Gierveld 3-item Loneliness Scale; F: Francie; G: Germany; N: Netherlands; R: Russia; B: Bulgaria; G: Georgia; J: Japan; EL: Emotional Loneliness; SL: Social Loneliness; GL: Global Loneliness; NL, No Loneliness; L: Loneliness; WHOQOL-Brief: the World Health Organization Quality of Life; SWLS: Satisfaction With Life Scale; VOL: Valuation of Life scale; R-UCLA: Revised University of California Los Angeles Scale; GDS-8: 8-item Geriatric Depression Scale; NA: Negative Affectivity (10-item subscale of the PANAS); PA: Positive Affectivity (10-item subscale of the PANAS); ULS-6: UCLA 6-item Loneliness Scale; MMSE: Mini Mental Sate Examination; LLS: Loneliness Literacy Scale; UCLA: University of California Los Angeles Scale; AUC: Area Under the Curve; DUKE-UNC: Social Support, Functional Social Support Questionnaire; T.I.: Turkish immigrants; M.I.: Moroccan immigrants; S.I.: Surinamese Immigrants; IS.I: Indo-Surinamese Immigrants; D: Dutch; ULS-16: UCLA 16-item Loneliness Scale; CRI: Composite Reliability Item; MSPSS: Multidimensional Scale of Perceived Social Support; IADL: Lawton Brody Instrumental Activities of Daily Living; BRCS: Brief Resilience Coping Scale; PWI: Personal Wellbeing Index; BRCS: Brief Resilient Coping Scale; PHQ-2: Patient Health Questionnaire-2; CVI: Content Validity Index; GLF: General Loneliness Factor; MF: Method Factor; FL: Familiar Loneliness; ML: Marital Loneliness; EC: Existential Crisis; IL: Intimate Loneliness; RL: Relational Loneliness; CES-D: Center for Epidemiologic Studies Depression Scale; GAI: Geriatric Anxiety Inventory
