# Supplementary figures and images for: A systematic review evaluating loneliness assessment instruments in older adults
Source: Front Psychol. 2023 Apr 25;14:1101462. doi: 10.3389/fpsyg.2023.1101462 (PMC10166865; doi:10.3389/fpsyg.2023.1101462)

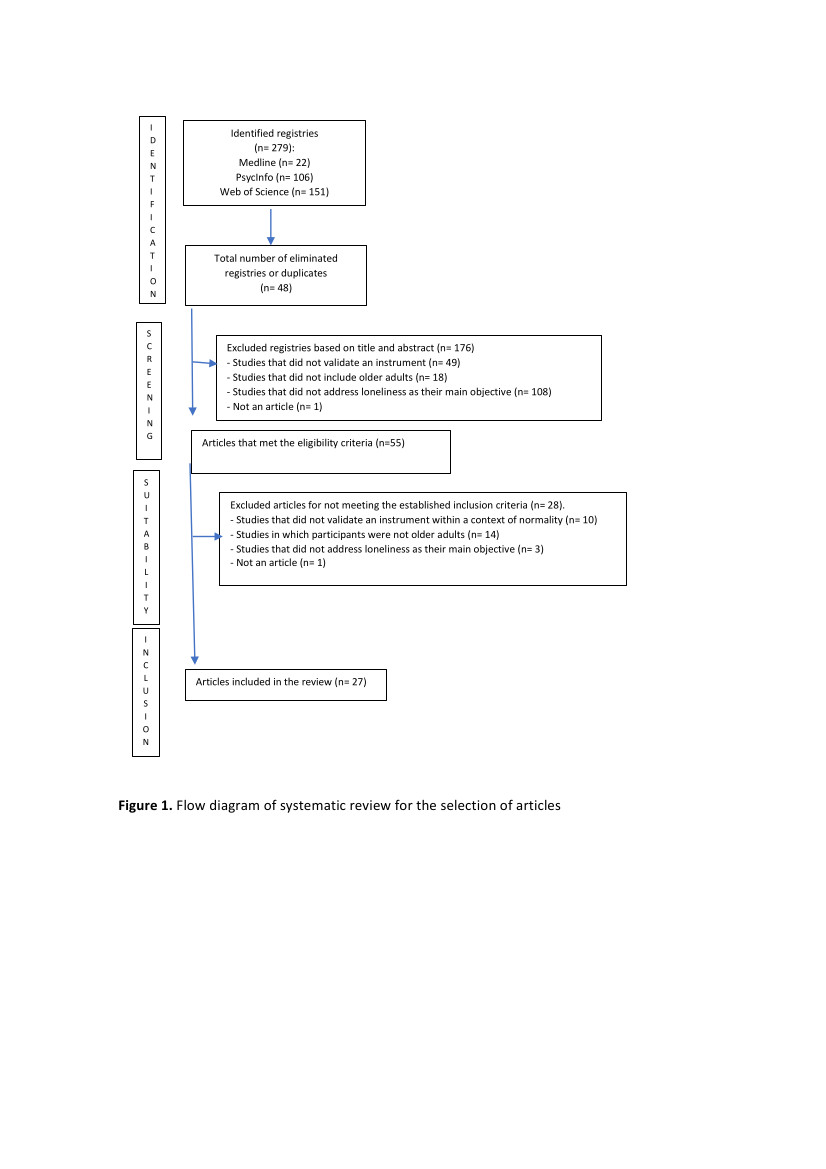

Supplement: Supplementary file 2 [file Image_1.jpeg]
